# Effects of Cadmium Sulfate on the Brown Garden Snail *Cornu aspersum*: Implications for DNA Methylation

**DOI:** 10.3390/toxics9110306

**Published:** 2021-11-15

**Authors:** Marius Georgescu, George Andrei Drăghici, Eliza-Florentina Oancea, Cristina Adriana Dehelean, Codruţa Şoica, Nicolae-Valentin Vlăduţ, Dragoș Vasile Nica

**Affiliations:** 1 Department of Functional Sciences, Physiology Discipline, “Victor Babeș” University of Medicine and Pharmacy Timișoara, Eftimie Murgu Sq. no. 2, 300041 Timișoara, Romania; georgescu.marius@umft.ro; 2 Center of Immuno-Physiology and Biotechnologies (CIFBIOTEH), “Victor Babeș” University of Medicine and Pharmacy Timișoara, Eftimie Murgu Sq. no. 2, 300041 Timișoara, Romania; 3 Faculty of Pharmacy, “Victor Babeș” University of Medicine and Pharmacy Timișoara, Eftimie Murgu Square No. 2, 300041 Timișoara, Romania; draghici.george.andrei@gmail.com (G.A.D.); codrutasoica@umft.ro (C.Ş.); 4 Research Center for Pharmaco-Toxicological Evaluations, Faculty of Pharmacy, “Victor Babes” University of Medicine and Pharmacy Timișoara, Eftimie Murgu Square no. 2, 300041 Timișoara, Romania; 5 Faculty of Pharmacy, “Vasile Goldiș” Western University of Arad, B-dul Revoluției no. 94, 310025 Arad, Romania; oancea.elizaflorentina@gmail.com; 6 INMA Bucharest, Bulevardul Ion Ionescu de la Brad 6, 077190 București, Romania; valentin_vladut@yahoo.com

**Keywords:** cadmium exposure, DNA methylation, 5-methylcytosine, metallothioneins, cadmium sulfate, CdMT gene, land snails, hepatopancreas

## Abstract

An extensive literature exists regarding the cellular, physiological, and genetic effects of cadmium (Cd)—A highly toxic, but commonly used trace metal in modern industry. However, limited data are available on its epigenetic effects, especially for terrestrial sentinel invertebrates. We determined Cd retention, total DNA methylation, and the methylation status of 5′ end of the *Cd-MT* gene in the hepatopancreas of the brown garden snail, *Cornu aspersum,* fed Cd sulfate for four weeks. Bodyweight changes and survival were also measured. Hepatopancreas cadmium increased in a dose-dependent manner from the third-lowest dose onward, with very large amounts being found for the highest treatment group. However, no mortalities occurred, irrespective of dietary Cd dose. We identified significant genome-wide hypermethylation in specimens given the highest dose, which overlapped with a significant bodyweight decrease. The *Cd-MT* gene showed an unmethylated 5′ end of the *Cd-MT* gene and this status was not affected by cadmium exposure. Hepatopancreas DNA methylation is as sensitive as bodyweight to non-lethal concentrations of dietary Cd given as cadmium sulfate but less responsive than tissue accumulation. Such an exposure event, by contrast, does not affect the methylation status of the *Cd-MT* gene 5′ end.

## 1. Introduction

Cadmium (Cd) is a non-essential trace metal classified as a global priority pollutant due to its environmental persistence, bio-accumulative nature, and high toxicity at low ambient concentrations [1,2,3]. Decades of toxicological research have yielded a compelling body of evidence for the cellular, physiological, and genetic implications of exposure to Cd and its compounds [2,4,5]. Such events are also known to affect epigenetic mechanisms shaping gene expression, including DNA methylation [6,7]. 5-Methylcytosine (5mC) is the best-characterized and most common form of DNA methylation, being linked to stable, long-term transcriptional silencing [8]. This evolutionary conserved epigenetic mark [9] occurs via covalent addition of a methyl (CH_3_) group to the 5′-carbon position of cytosine pyrimidine ring primarily found in cytosine-guanine (CpG) dinucleotides [10]. Global and gene-specific changes in 5mC content associated with Cd exposure have been described in most vertebrate classes [11,12,13,14,15]. However, limited information exists for terrestrial invertebrates routinely used as sentinel organisms, such as annelids [16] or land snails [7]. The latter species are pivotal for terrestrial ecosystem functioning, serving as key players in calcium cycling and acting on trophic level, both as herbivorous and detritivore organisms [17,18].

CpG islands are often located at the 5′-end of genes, with promoters and transcription start sites embedded within these genomic regions of high CpG dinucleotide densities [19,20]. The methylation status of these gene-regulatory elements can play critical roles in modulating vertebrate gene expression, especially for inducible genes responsive to various biotic and abiotic environmental factors [20]. Cadmium exposure has been associated with altered patterns of DNA methylation at the 5′-end of vertebrate genes [21,22]. For most invertebrates, by contrast, there is only limited information regarding the occurrence of cytosine methylation at the 5′-region of genes and the effects of Cd on DNA methylation [20,23,24]. Of particular importance in the context of cadmium and terrestrial gastropods is to identify the methylation status in the 5′-region of the Cd-selective metallothionein (Cd-MT) gene and to determine whether such an exposure event can affect it. Unlike constitutively expressed genes with housekeeping functions, the Cd-MT gene is inducible and pivotal for cadmium detoxification in many species of land snails, including the brown garden snail, *Cornu aspersum* (Müller 1774)—an appropriate invertebrate model for investigating biological responses to cadmium [7,25,26,27].

To the best of our knowledge, only one study to date has investigated the effect of Cd on gastropod methylome. When given as cadmium chloride (CdCl_2_), dietary Cd affected the global 5mC content of genomic DNA in the hepatopancreas of mature *C. aspersum* snails [7]. Different anions/cations could exert different methylomic effects [28], but the impact of other cadmium compounds on epigenetics in this specific snail and other gastropod species is still not clear. These data are important for broadening our understanding of Cd ecological risk, especially for compounds commonly used in modern industry, but with less studied epigenotoxic profiles. Such an example is cadmium sulfate (CdSO_4_), a key compound in the manufacture of fluorescent screens, vacuum tubes, transistors, and photovoltaic and solar cells; as well as a pigment in paints and inks [3].

Experimental data also showed that the *Cd-MT* gene transcription is initiated after Cd exposure and shows a persistent induction rate [26]. However, the methylation status of the 5′ end of this gene or the effect of Cd on this gene segment has not been yet examined. At least two important questions derive from the foregoing considerations: (1) Whether and to which extent does cadmium sulfate exposure modify global 5mC levels of *C. aspersus* hepatopancreas cells? (2) What is the relevance of the methylation status of CG pairs at the 5′ region of the *Cd-MT* gene as an endpoint of Cd exposure. To test these hypotheses, mature specimens of *C. aspersum* were maintained under laboratory conditions for four weeks and given Cd-spiked diets at doses covering a wide concentration range, including the 0.2 milligrams per kilogram fresh weight benchmark level allowed in vegetal foods regularly eaten by terrestrial gastropods, such as fruits and leafy vegetables [29,30]; and those for Cd toxicity on key cellular endpoints in these invertebrates [7,31]. Genome-wide DNA methylation, methylation of the 5′-UTR of the *Cd-MT* gene, and Cd levels in the hepatopancreas of *Cornu aspersum* were determined. Bodyweight changes and snail death rates were used as a sublethal endpoint and a lethal endpoint, respectively. Our findings are important because they not only expand previous knowledge about the effect of Cd on an ecologically relevant study system but also fill a gap concerning stress-dependent changes in methylation patterns in gastropod species.

## 2. Materials and Methods

### 2.1. Landsnail Origin, Maintenance and Exposure

*Cornu aspersum* is one of the most widely used gastropod models in ecotoxicological studies because it can be easily maintained under laboratory conditions and is commercially available [7]. A total of 180 adult specimens (with a reinforced lip at shell aperture) were obtained from the “Mokry Dwór” snail farm (Krzymów, Wielkopolska, Poland). The snails were kept in groups of 10 in 20 L aerated polypropylene containers (50 × 20 × 20 cm) with perforated side walls at 20 °C in a 12/12 light/dark cycle for four weeks prior to the beginning of the experiments. The container bottom was covered with a layer of ash-free filter paper (50 × 20 cm), which was wetted twice daily with double distilled water. Exposure experiments were conducted under controlled laboratory conditions similar to those used in our previous investigations, using Cd-spiked, agar-based diets at concentrations between 0 and 100 mg per kg dry weight (mg/kg d. wt) [1,7]. Hepatopancreas samples were taken at the end of the exposure period to assess the effect of Cd on genome-wide 5mC levels and the methylation status of the *Cd-MT* gene. All experiments were performed at the Laboratory Animal Facility from the “Victor Babes” University of Medicine and Pharmacy from Timisoara.

At the start of the exposure experiments (September 2018), the most homogeneous 120 specimens were sorted based on their size, as determined via measuring the shell height (with a digital caliper) and the snail weight (with an analytical balance). The mean shell size of selected gastropods was 2.95 ± 0.34 cm in length, whereas the mean weight was 8.79 ± 1.85 g. The snails were given *ad libidum* Cd-spiked diets for four weeks. The eight nominal Cd treatments used to prepare the agar-based diets were 0, 0.05, 0.1, 0.2, 1, 10 and 100 mg/L (abbreviated as Cd0, Cd0.05, Cd0.1, Cd0.2, Cd1, Cd10, and Cd100). In contrast to our previous studies [1,4,7], cadmium sulfate (CdSO_4_ ≥ 99.99% trace metal basis(Sigma-Aldrich, St. Louis, MO, USA) was used here as a source of cadmium instead of cadmium chloride.

Exposures were run in two independent experiments per treatment group (Replica I and Replica II), with each replica consisting of 10 snails. Body weight was measured at the start and the end of the 4-week experimental period for four individuals per replicate jar. Four specimens for each experimental group (i.e., two specimens per replicate) were then dissected. This relatively modest sample size is not unusual for global DNA methylation studies due to cost constraints. In addition, our previous work showed that even smaller sample sizes, that is three specimens for each treatment group, can be used to detect Cd-related changes in total 5mC content in DNA of the hepatopancreas of adult *C. aspersum* [7]. The soft body was removed from the shell with a hemostat to collect hepatopancreas samples. These samples were used for performing chemical analyses and extracting DNA. Weekly food samples were randomly taken to check the feed cadmium concentration.

### 2.2. Snail Fitness Parameters

Snail fitness was assessed by determining the weight gain and survival rates. Weight gain was calculated as the difference between the snail weight at the start and the end of the exposure period. All snails were weighed with an analytical balance to the nearest 0.01 mg.

### 2.3. Chemical Analyses

Hepatopancreas cadmium concentrations were assessed as previously described [1,4,7]. In short, the frozen samples (*n* = 4 for each treatment) were thawed and oven-dried at 105 °C for 24 h. After being weighed and calcinated, these samples were dissolved by wet digestion with 65% nitric acid (Merck KGaAe) and heated on a hot plate to dryness. The ash was dissolved in 20 mL of 0.5 N HNO_3_, filtered through ash-free filter paper and the filtrate volume was brought to 30 mL with 10 mL HNO_3_ 0.5 N. A similar approach was used for determining Cd levels in food samples, with triplicate samples per each treatment being analyzed at one, two, and three weeks.

Measurements were performed via flame (air-acetylene) atomic absorption spectrometry using a VARIAN AA240FS (Agilent, Santa Clara, CA, USA) fitted with a Cd-specific hollow-cathode lamp as a source of radiation. The results were expressed as milligram per kilogram dry weight (mg/kg d. wt). Standard solutions (1000 mg/L) of Cd-ICP multi-element standard solution IV CertiPUR were purchased from Merck. Only pure double distilled water was used to prepare the reagents and standard solutions. The cadmium quantification limit was 0.01 mg/kg d. wt. It was determined via the calibration curve used to validate the accuracy of the method by measuring the standard reference material obtained from the China National Analysis Center for Iron and Steel (NCS Certified Reference Material85105a). The average percent recovery was 98%, with the corresponding variation coefficients being less than 6%. The spectrometer was recalibrated every 10 samples, and blank samples were run every 15 samples. Blank samples were prepared in a similar manner to the aforementioned samples, using pure double distilled water instead of standard solutions.

### 2.4. DNA Extraction and 5mC Quantification Analysis

**Figure 1:**
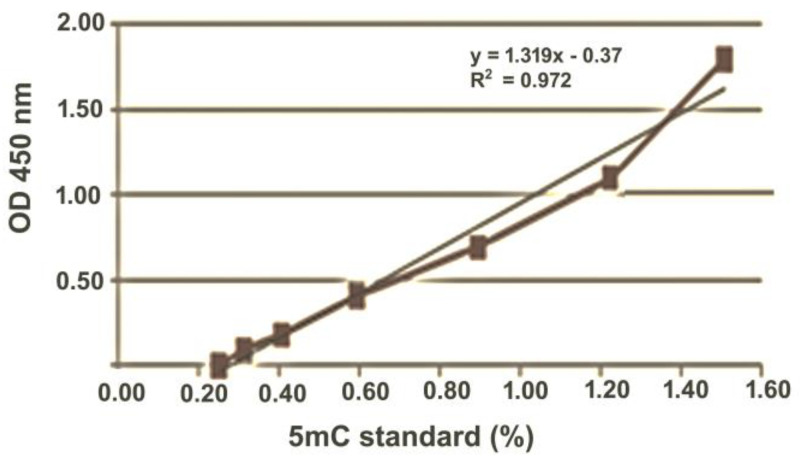
ELISA standard curve. The upper right shows the standard curve equation and the corresponding coefficient of determination. The *x* axis illustrates the 5mC values of the negative control (0%) and the positive controls (0.1%, 0.2%, 0.5%, 1%, 2% and 5%) expressed as ln (5mC% + 1), whereas the *y*-axis shows the corresponding absorbances.

Genomic DNA was isolated from hepatopancreas samples using the GenElute™ Mammalian Genomic DNA Miniprep Kit (Merck KGaA, Darmstadt, Germany) according to the manufacturer’s directions. The DNA purity was checked by UV absorption (260/280 nm, DS-11 Spectrophotometer, DeNovix, Wilmington, NC, USA) [7]. Total 5mC DNA level was quantified using the MethylFlash Global DNA Methylation (5mC) ELISA Easy Kit (EpiGentek, Farmingdale, NY, USA). This kit provides a fast and precise method to determine global DNA methylation changes, with a detection limit of 5 nanograms (ng) of fully methylated DNA. In addition, the use of such ELISA-based assays for serial measurements of 5mC can serve as a cost-effective and reliable alternative to commonly used methods for whole-genome DNA methylation quantitation, such as chromatography, radioactive filter-binding, or bisulfite sequencing-based methods [32,33]. Optical density (OD) was measured at a wavelength of 450 nanometers (nm) with a Stat Fax 4200 microplate reader (Awareness Technology, Palm City, FL, USA). For absolute 5mC quantifications, a standard curve was created by graphing the corresponding ODs versus the log_10_-transformed concentrations of the positive controls. The coefficients of determination (R^2^) for ELISA calibration curves were above 0.97; an example is shown in Figure 1.

### 2.5. Methyl Specific Polymerase Chain Reaction (MSP)

Until now, only a 1128-base-pair sequence of the *Cd-MT* gene promoter of *Cornu aspersum* is known. This gene segment includes the metal responsive element binding sites (MREs), which are central to metal-dependent upregulation of MT gene expression in vertebrate species [34]. However, it is not known how far the promoter region may extend upstream beyond the known sequence. The cDNA sequence of the *C. aspersum* hepatopancreas encoding the entire Cd-MT protein (GenBank: EF152281.1) was hence used to manually design suitable primers for methylation analysis by bisulfite sequencing. The 5′-UTR sequence of the Cd-MT cDNA contains several CpG sites upstream of the coding ATG. Initially, one forward primer (CdMTbs-F1/ggatttatYGtaggatattaattaagg, promoter) and two reverse primers (CdMTbs-R1/ctaaaaataaaaccaaataccaatcctac, position 361; CdMTbs-R2/Ccttaccacacttacaaccatc, position 300) were designed to determine optimal bisulfite PCR conditions. Details regarding the CpG and GpC sites analyzed are given in Figure S1 (Supplementary Material). Bisulfite conversion of genomic DNA isolated from hepatopancreas was performed using the ‘high molarity’ protocol as previously described [35]. Typically, PCRs were run in a 40 µL reaction volume, with ~0.25–0.5 µg of bisulfite converted DNA using the HotStarTaq Master Mix reagent (Qiagen, Hilden, Germany), following the specifications of the supplier’s manual. A 10 µL aliquot of the PCR product was run on a 1.5% TAE (Tris-acetate-EDTA)-agarose gel to check for specific amplification. The amplicons were visualized via ethidium bromide staining following agarose gel electrophoresis (Merck, Darmstadt, Germany)

### 2.6. Statistical Analysis

Genome-wide DNA methylation in the hepatopancreas of brown garden snails, *C. aspersum*, were analyzed using a one-way ANOVA. Before conducting ANOVA, the metal and DNA methylation data sets were log_10_-transformed and then checked for normality and heteroscedasticity using Anderson-Darling tests and Bartlett’s tests, respectively. If the ANOVA results were significant, posthoc analysis was performed against controls using Dunnet’s tests. The same statistical approach was used for analyzing the effect of dietary cadmium on absolute values for weight gain/loss. This parameter was expressed as the difference between the measured values of the bodyweight at the end and the start of the experiment. Finally, a Chi^2^ test was conducted on a 7 × 2 contingency table to compare snail survival across different treatment groups, with tests based on 2 × 2 contingency tables being applied against controls in case of significant differences. All statistical analyses were performed using Statistica version 8 (StatSoft Inc., Tulsa, OK, USA). Statistical significance was defined at *p* less than 0.05.

## 3. Results

The mean levels of Cd in fodder samples were (*i*) for the Cd0 treatment, below the detection limit (0.01 mg/kg d. wt); (*ii*) for the Cd0.05 treatment, 0.05 ± 0.02 mg/kg d. wt; (*iii*) for the Cd0.1 treatment, 0.11 ± 0.04 mg/kg d. wt; (*iv*) for the Cd0.2 treatment, 0.19 ± 0.05 mg/kg d. wt; (*v*) for the Cd1 treatment, 1.07 ± 0.23 mg/kg d. wt; (*vi*) for the Cd10 treatment,10.79 ± 4.55 mg/kg d. wt; (*vii*) for the Cd100 treatment, 98.87 ± 8.99 mg/kg d. wt. The consumption rates were not determined, but 3 weeks after the start of the experiment most specimens from the highest treatment group tended to avoid food and enter into aestivation.

### 3.1. Hepatopancreas Cadmium

**Figure 2:**
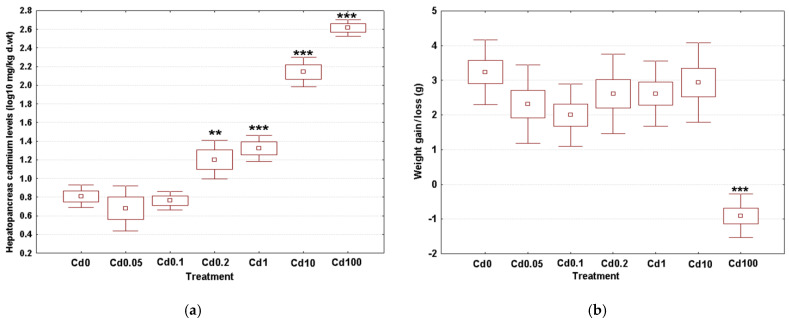
Effect of dietary cadmium on (**a**) hepatopancreas cadmium levels (*n* = 4) and (**b**) body weight gain/loss (*n* = 8). Metal data are shown on a log_10_ scale, whereas weight data are given as absolute values. Data sets are depicted as mean (point) with one standard error (box) and one standard deviation (error bar) (Dunnet’s tests, ***—*p* < 0.001, **—*p* < 0.01, *—*p* < 0.05).

Average concentrations of hepatopancreas cadmium are shown in Figure 2a. The measured values varied between 5.31 ± 2.59 mg/kg d. wt in controls and 419.70 ± 84.87 mg/kg d. wt in the highest treatment group (Cd100) (Figure 2a). The log_10_-transformed data sets were normally distributed (Anderson-Darling tests, *p* ≥ 0.472) and showed similar variances (Bartlett’s test, *p* = 0.646). Dietary dose had a significant influence on Cd retention in the hepatopancreas (ANOVA, (F(6, 27) = 87.89, *p* < 0.001). Post hoc testing using the Dunnet’s approach revealed that the measured values increased significantly (Figure 2a starting from the third-lowest dose (Cd0.2) onward.

### 3.2. Fitness Parameters

#### 3.2.1. Body Weight Gain

The mean values of body weight gain (loss) are given in Figure 2b. The average weight at the start of the experiment was between 8.20g in the snails exposed to the third-highest dose (Cd1) and 9.82g in the specimens from the highest treatment group (Cd100). At the end of the experiment, the weight gain/loss ranged from -0.90g in gastropods given the highest Cd dose (Cd100) up to 3.23g in controls. The data sets related to the later variable were normally distributed (Anderson-Darling test, *p* ≥ 0.209) and homoscedastic (Bartlett’s test, *p* = 0.793). We identified a significant effect of dietary Cd uptake on weight gain (ANOVA, (F(6, 55) = 12.87, *p* < 0.001). Post hoc analyses using the Dunnet’s procedure revealed a similar weight gain compared to controls for most treatment groups (Figure 2b). The only exception was noted for the gastropods fed the highest Cd dose (Cd100), which showed a significant 10% reduction in body weight.

#### 3.2.2. Snail Survival

No mortalities were observed both for Cd-fed individuals and controls. Statistical analysis revealed no significant difference in survival among different groups (Chi^2^ test, *p* = 1). Therefore, the survival of mature snails, *C. aspersum*, appears not to be affected by continuous four-week exposure of up to 100 mg/kg d. wt Cd in the food.

### 3.3. Genome-Wide DNA Methylation in the Hepatopancreas

**Figure 3:**
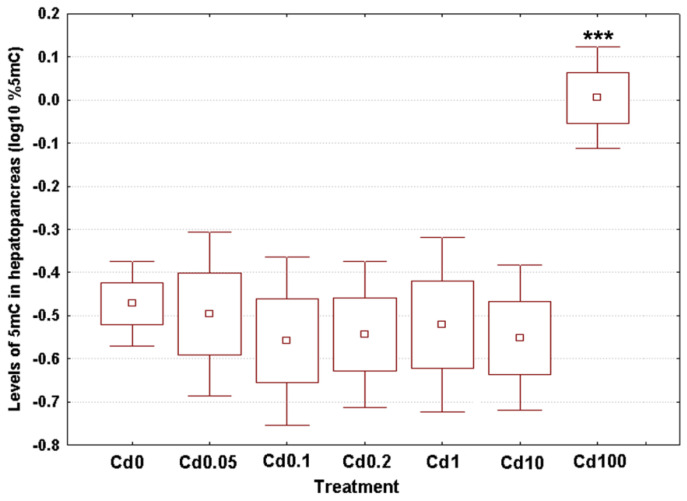
Effect of dietary cadmium on genome-wide 5mC levels in DNA of the hepatopancreas of *C*. *aspersum* adults (*n* = 4). Data are shown on a log_10_ scale as mean (point) with one standard error (box) and one standard deviation (error bar) (Dunnet’s tests, ***—*p* < 0.001, **—*p* < 0.01, *—*p* < 0.05).

Mean 5mC levels are depicted in Figure 3. The measured values ranged from 0.29% in the snails exposed to the lowest Cd dose (Cd0.05) up to 0.99% in the specimens from the highest treatment group (Cd100), with an overall weighted mean for all groups of 0.40% (Figure 3). These data sets met the assumptions of normality (Anderson-Darling tests, *p* ≥ 0.189) and homogeneity of variance (Bartlett’s tests, *p* = 0.912). Cadmium exposure was associated with significant changes in DNA methylation levels (ANOVA, (F(6, 27) = 5.89, *p* = 0.001). Post hoc testing using the Dunnet’s approach revealed significant hypermethylation for gastropods consuming the highest Cd dose (Cd100), but no effect was identified for the other treatment groups (Figure 3).

### 3.4. Methylation Status of CG Pairs at the 5′ Region of the Cd-MT Gene

In the case of controls, all cytosines from the seven analyzed *CpG* sites were converted to thymines after bisulfite treatment and PCR amplification, irrespective of the primer pairs used. Identical results were obtained for specimens given different doses of cadmium in the diet for 28 days. These data demonstrate that the 7 CpG sites at the 5′-UTR of the Cd-MT mRNA are unmethylated. The 5′ region close to the presumed *Cd-MT* transcription start site is not constitutively methylated and the methylation status does not appear to be influenced by cadmium exposure.

## 4. Discussion

We analyzed the effect of environmentally relevant concentrations of dietary cadmium (given as cadmium sulfate) on terrestrial ecosystems using the brown garden snail, *C. aspersum*, as an invertebrate (eco) toxicological model. The accumulation of this non-essential trace metal into the hepatopancreas of mature specimens started to increase significantly from doses as low as 0.2 mg/kg d. wt. This trend is comparable with those reported in studies using specimens of similar age, but cadmium chloride as a source of dietary Cd [1,7,36]. Hence, it appears that chloride and sulfate ions may affect in a similar manner the dynamics of cadmium retention in the hepatopancreas of mature snails, *C. aspersum*.

Cadmium exerted a significant effect on body weight only in adult individuals from the highest treatment group. For comparable exposure doses and times, several studies with younger specimens revealed a significant dose-dependent decrease or higher reductions of body weight (>35%) [36,37,38]. These divergent results could be attributed to an age-dependent decrease in metabolic rate [39] leading to higher gastrointestinal adsorption of Cd, and subsequently its elevated retention in the hepatopancreas [2].

All snails survived throughout the four-week experimental period, irrespective of the Cd dose administered. These results are in agreement with data from previous investigations [1,40,41] and attest to the exceptional ability of mature gastropods, *C. aspersum*, to tolerate substantial amounts of dietary cadmium without notable lethal effects. Low death rates (0% to 6.7%) were also identified in younger specimens given Cd-enriched diets [36,37,38,42]. Overall, these data do not render mortality or bodyweight of adult gastropods, *C. aspersum*, as ecologically relevant endpoints for assessing the hazardous effects of environmental cadmium over a one-month period.

The range of genome-wide DNA methylation levels observed in the present study is close to those determined using a similar ELISA-based approach in mature brown garden snails exposed to dietary cadmium as cadmium chloride [7], or in adult specimens of acute bladder snail, *Physella acuta* (Draparnaud, 1805), and southern creeper, *Zeacumantus subcarinatus* (G. B. Sowerby II, 1855), that is between 0.2% and 0.9% [43,44]. Only the gastropods fed the greatest Cd dose as cadmium sulfate exhibited marked differences compared to controls, revealing significant hypermethylation of hepatopancreas DNA at 28 days (week 4). This is in contrast with methylation data available for cadmium chloride [7]. Thus, individuals reared under identical exposure conditions (snail age, exposure duration, rearing conditions) showed global cytosine hypermethylation at doses of 10 mg/kg d. wt Cd and higher [7]. Hence, the effect of Cd on 5mC levels in hepatopancreas DNA seems to be influenced by the type of inorganic anion bound to Cd. Indeed, there is evidence that different inorganic salts of the same metal can have different effects on genomic DNA methylation levels [28]. In the hepatopancreas DNA of *C. aspersum*, cytosine methylation seems to be constitutively absent at the 5′-end of the *Cd-MT* gene. The 5′-UTR of the *Cd-MT* mRNA is likely located close to the promoter and our data suggest that the *Cd-MT* gene in this snail species is not regulated by this type of epigenetic mechanism. We also found that cadmium uptake via food had no effect on the methylation status of the Cd-MT gene 5′-region. Consistent with our findings, Drechsel et al. observed no methylation in CG pairs of *the* Cd-inducible metallothionein gene *wMT*-2 promoter from the common earthworm, *Lumbricus terrestris* (Linnaeus, 1758), for both Cd-exposed and control individuals [45]. However, we cannot exclude a potential effect of Cd on the DNA methylation status of promoter or introns (non-coding regions) without examining in detail these regions of the *Cd-MT* gene. With this in mind, the methylation status of MREs and adjacent areas in the *Cd-MT* promoter region warrant future investigations, given the importance of these gene segments as binding sites for metal-response element-binding transcription factors (MTFs) [34]. This topic is of interest not only for *C. aspersum*, but also for other commonly used gastropod models, such as the Roman snail, *Helix pomatia* (Linnaeus, 1758). Both species share MRE binding sites, including CG pairs in their *Cd-MT* gene promoter region, but their *Cd-MT* genes show distinct transcription patterns. These variable expression patterns appear to be partly related to structural differences in their respective *Cd-MT* gene promoter regions, like the variable number of MRE binding sites [34]. Their MTF-2 nucleotide sequence contains CG pairs [34], but their methylation status is yet to be determined. Another topic to be addressed in future studies is the impact of cadmium on methylation of the gene body due to its important role in modulating stress and environmental responses in many invertebrates [19], including mollusks [46].

Based on the current results, the global 5mC content of genomic DNA in hepatopancreas tissue appears to be a less sensitive biomarker of cadmium exposure compared to hepatopancreas cadmium level. However, this endpoint was as sensitive as body weight to non-lethal concentrations of dietary cadmium given as cadmium sulfate. Moreover, significant hypermethylation of hepatopancreas DNA was associated with a significant decrease in body weight and snail tendency to enter into aestivation, with all these events occurring only for the highest treatment group. In the sea cucumber *Apostichopus japonicus*, the aestivation-induced intestinal hypometabolism was found to be driven by transcriptional suppression of metabolic pathways mediated via DNA hypermethylation [47]. In the near relative species *Helix pomatia*, the *Cd-MT* gene expression is not only upregulated following Cd exposure but can also vary owing to desiccation, where gastropods switch into aestivation [48,49]. The changes observed here in total 5mC levels in hepatopancreas tissue may hence be, at least partly, connected to Cd-induced lethargy, and not necessarily indicate a direct effect of cadmium on the hepatopancreas DNA methylation. More precisely, Cd exposure is perhaps not the main cause for hepatopancreas DNA hypermethylation, but rather aestivation due to Cd exposure.

The main limitation of this study is the fact that we did not investigate the effect of Cd on DNA methylation profiles and/or expression of genes relevant to such an exposure event, e.g., the key genes of the DNA methylation machinery (related to DNA methyltransferases, Ten Eleven Translocation enzymes, or Methyl-CpG-binding domain proteins). By using RT-PCR and primers specific to the mammalian DNMT genes, however, we have recently found pertinent evidence for the presence of the DNMT1 gene, but not the DNMT3 genes in the *C. aspersum* genome [50]. This gene is central for the maintenance of DNA methylation in eukaryotes [20]. The candidate gene identified by us appears to be functionally expressed and sensitive to Cd exposure. Unfortunately, the genome of *C. aspersum* is yet to be sequenced and only a part of its transcriptome was characterized [51]. In fact, such high-resolution analysis is currently prohibited not only for this species but also for other terrestrial mollusks due to the limited number of reference genomes available [51] and little information existing about their methylome [9]. Moreover, the snail hepatopancreas contains different cell types [31], which may exhibit different DNA methylation levels and profiles. Therefore, future studies should aim to generate genome-wide and gene-specific, high-resolution DNA methylation profiling and conduct functional analysis of 5mC and other DNA methylation variants on individuals and different species of land snails, with emphasis on cell type, environmental status, and ontogenetic stage. Such fundamental knowledge is a *sine qua non* prerequisite for fully determining the impact of Cd on the hepatopancreas DNA methylome and deciphering the mechanisms underlying its effect.

## 5. Conclusions

To summarize, the results show that genome-wide hepatopancreas DNA methylation is a less sensitive biomarker of Cd exposure compared to tissue accumulation, but as responsive as body weight for non-lethal concentrations of dietary cadmium given as cadmium sulfate. In addition, our findings provide an indication that cytosine methylation could be constitutively lacking at 5′-end of the *Cd-MT* gene, and this status is independent of the effect of Cd exposure. Overall, the data obtained fill a gap concerning stress-dependent changes in methylation patterns in gastropod species and improve the current state of knowledge linking environmental pollutants and gastropod methylome.

## Supplementary Materials

The following are available online at https://www.mdpi.com/article/10.3390/toxics9110306/s1, Figure S1: CpG and GpC sites (double-stranded) in the the 5′-UTR of the *Cd-MT* mRNA in *C**. aspersum.*
