# Supplementary material for: Effects of Cadmium Sulfate on the Brown Garden Snail Cornu aspersum: Implications for DNA Methylation
Source: Toxics. 2021 Nov 15;9(11):306. doi: 10.3390/toxics9110306 (PMC8619149; doi:10.3390/toxics9110306)
Supplement: Supplementary file 1 [file toxics-09-00306-s001.zip › toxics-1415923-supplementary.pdf]

toxics

MDPI

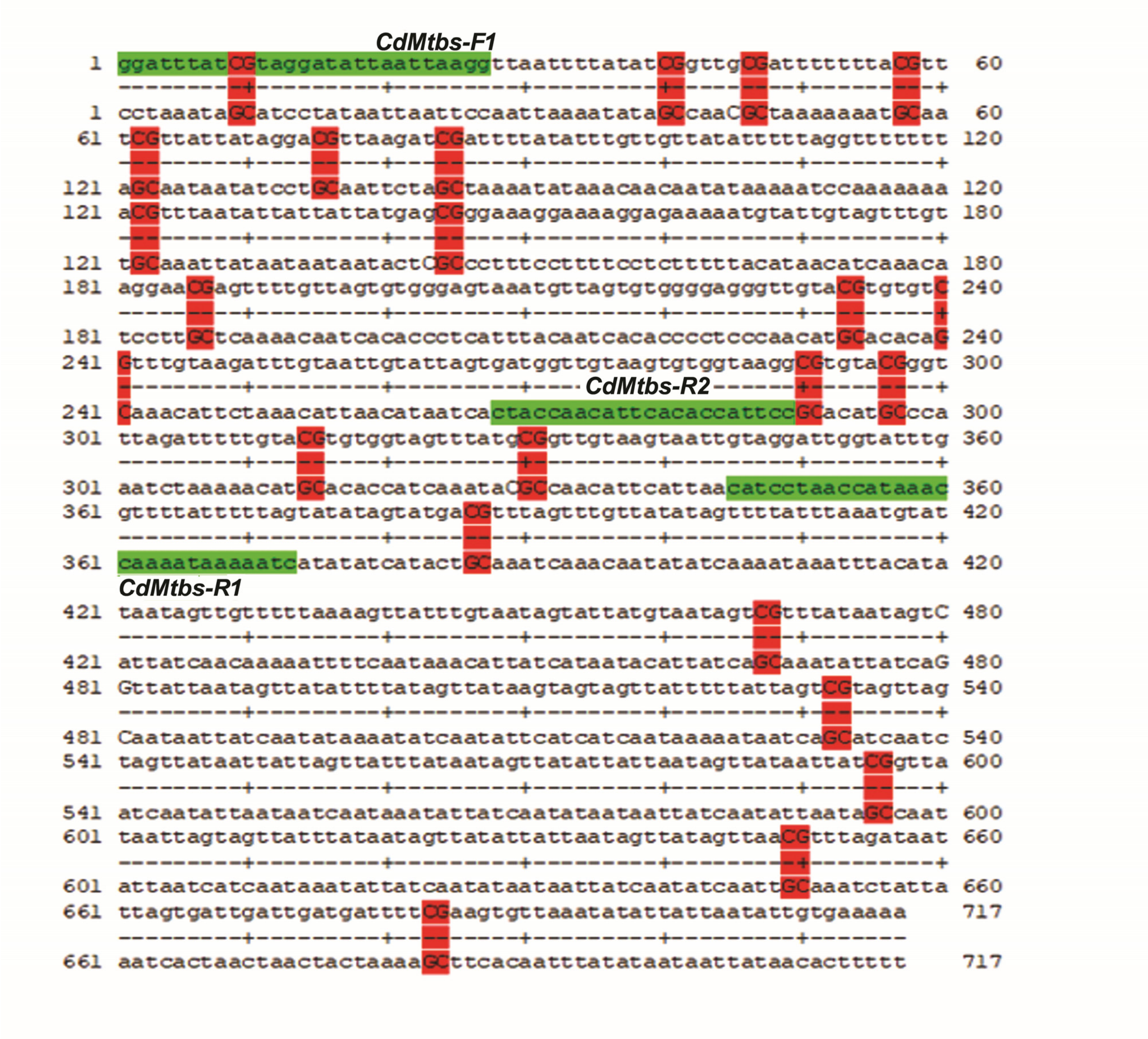

Figure S1. CpG and GpC sites (double-stranded) in the 5′-UTR of the *Cd-MT* mRNA in *C. aspersum*. Nucleotid sequence was obtained with Gene Runner based on the amino acid sequence alignment of *Cd-MT* protein. The CpG and GpC sites are highlighted in red. The primers sequences are highlighted in green.
